# Blood Donors' Age, Haemoglobin Type, G6PD Status, and Blood Group Impact Storability of CPDA-1 Banked Whole Blood: A Repeated-Measure Cohort Study in Cape Coast, Ghana

**DOI:** 10.1155/2020/4959518

**Published:** 2020-05-30

**Authors:** Patrick Adu, Gilbert Appiah Kubi, Amos Kumi, Raphael E. K. Gbedoho, Festus Ansah Kwakye, Emmanuel Sarpong, Constantine Drai, Samuel Dompreh, Fredrick Afful Sersah, Eric Ofori Gyamerah

**Affiliations:** ^1^Department of Medical Laboratory Science, School of Allied Health Sciences, University of Cape Coast, Cape Coast, Ghana; ^2^Department of Biochemistry, School of Biological Sciences, University of Cape Coast, Cape Coast, Ghana; ^3^Department of Molecular Biology and Biotechnology, School of Biological Sciences, University of Cape Coast, Cape Coast, Ghana

## Abstract

**Background:**

The high prevalence of haemoglobin variants and glucose 6-phosphate dehydrogenase disorder (G6PDd) in sub-Saharan Africa means that substantial proportions of donor blood units carry these red cell abnormalities.

**Aim:**

This study investigated the impact that inherited haemoglobin variants and/or G6PD status have on whole blood banked at 4–6°C for 35 days.

**Method:**

This repeated-measure cohort study was undertaken on 103 donor blood units collected into blood bag containing CPDA-1 anticoagulant. On days 0, 7, 14, 21, and 35, full blood count, osmotic-induced haemolysis, and plasma K+ levels were estimated. Also, on day 0, G6PD status, haemoglobin variants, % foetal haemoglobin, and blood group of donor units were determined using methaemoglobin reductase, cellulose acetate electrophoresis, modified Bekte alkali denaturation assay, and slide haemagglutination test, respectively.

**Result:**

Overall, although plasma K+ levels increased during storage, donor units from individuals ≥20 years, G6PD normal, Hb AC, or blood group B had comparatively higher percentage change in plasma K+ during storage. Osmotically induced haemolysis of donor units was significantly decreased in Hb AC (compared with Hb A or AS) donor units on days 7, 14, 21, and 35 (*p* < 0.0001 in each case). G6PDd donor units had comparatively reduced osmotic-induced lysis compared with G6PD normal units, reaching a statistical significance on day 35 (*p* = 0.043). Also, Hb AC units had comparatively nonstatistically higher plasma K+ at all time points (compared with Hb A or AS). Furthermore, whereas donor units from individuals ≥20 years showed significantly higher median free haemoglobin on day 21 (compared to donor <20 years), when donor units were stratified per Hb variants, only Hb AS units had median free haemoglobin below the 0.8% threshold after 35 days' storage.

**Conclusion:**

Age of donor, blood group, Hb AC variant, and G6PD status may be important considerations in the storability of whole blood.

## 1. Introduction

In sub-Saharan Africa, blood transfusions are usually in response to anaemia caused by malaria, haemoglobinopathies, and road-traffic accidents. To assure ready availability of blood products, blood banking is an essential component of the health care delivery. However, it has been shown that inherent blood donor characteristics such as genetics and/or lifestyle choices impact the donor variation effect to donor blood units during storage [1, 2]. Since haemoglobinopathies and red cell enzymopathies abound in sub-Saharan Africa, individuals who are heterozygous for these red cell abnormalities may inadvertently donate blood. For example, previous research works have demonstrated that 11.3% to 19.5% of blood donors in Ghana [3, 4] and 26.1% in Nigeria [5] carried the sickle cell trait. Also, 19.5–25.5% of blood donors have been estimated to be carrying qualitative glucose 6-phosphate dehydrogenase deficiency (G6PD) disorder in the subregion [4, 5]. In spite of the estimated high prevalence of inheritance of sickle cell trait and G6PD deficiency among blood donors in the sub-region, there is paucity of data on how these inherited donor characteristics influence red cell storage lesion as most of the previous studies were cross-sectional studies. Herein, using % free haemoglobin, osmotic-induced red cell lysis, and plasma potassium as a measure of whole blood storage lesion, we sought to determine the effect of blood donors' G6PD status, haemoglobin type, age, percentage foetal haemoglobin, and blood group on whole blood units stored in citrate phosphate dextrose 1 (CPDA-1) for 35 days (at 2°C–6°C).

## 2. Methods and Materials

### 2.1. Study Design/Study Site

This repeated-measure laboratory-based cohort study was carried out from February 2019, to May 2019, at the Cape Coast Teaching Hospital (CCTH) in the Cape Coast Metropolis, Ghana. The study population included individuals who voluntarily donated blood during a mobile session at the Adisadel College. As per the existing donor recruitment protocol of the Ghana Health Service, blood donors (≥16 years) who tested negative for all the transfusion-transmitted disease screening assays such as HIV, syphilis, and Hepatitis B and C virus were enrolled. A total of 103 donor (aged 16–47 years) samples were collected for the study using a convenience sampling technique.

### 2.2. Sample Size Calculation

The sample size was determined by the confidence interval formula (*Z*^2^*p*(100 − *p*))/*d*^2^  [6], where z = standard error (1.96) associated with 95% level of confidence. The co-inheritance of both G6PD deficiency and sickle cell haemoglobin trait among donors in Berekum, Ghana, is 7% [4]. Thus, *p* = estimated percent in the population (7%) = 0.07; *q* = 100−*p* = 93 = 0.93; *d* = acceptable sample error (5%) = 0.05. Hence, the required minimum sample size was 100; however, a total of 103 donor blood units were used for this study.

### 2.3. Questionnaire

Semi-structured questionnaires were used to capture demographic data, medication history, lifestyle choices (smoking and intake of alcoholic beverages), donor type and history, and other pertinent information of each participant.

### 2.4. Experimental Protocols

#### 2.4.1. Specimen Collection and Storage

About 450 ml of participant's blood sample was collected into CPDA-1 anticoagulated blood bag (Blue Arrow, China) by the hospital phlebotomists. For the purpose of the study, 7 ml of the CPDA-1 anticoagulant was allowed into the sample pouch attached to the main blood bag, which was filled with the initial 50 ml of whole blood. The rest of the whole blood was directed into the main blood bag. The 50 ml whole blood in the sample pouch was stored at 4°C for 35 days. 10 ml samples were aspirated aseptically on days 0, 7, 14, 21, and 35 for haematological and biochemical assays.

#### 2.4.2. Blood Grouping

On the zeroth day of donor's blood collection, ABO blood grouping of each sample was undertaken using monoclonal anti-A and anti-B (Lab-Care Diagnostic, India) in accordance with manufacturer's protocols. A slide method was used to observe haemagglutination reactions formed during the procedure.

#### 2.4.3. Qualitative Glucose 6-Phosphate Dehydrogenase Deficiency Test

The methaemoglobin reduction test previously described [7] was used to qualitatively assay for G6PD status on the zeroth day of blood collection. For each sample, three tubes were set: test (*T*), normal (*N*) control, and deficient (*D*) control; the *N* and *D* tubes were included as controls to validate the results.

#### 2.4.4. Cellulose Acetate Electrophoresis (pH 8.4)

The haemoglobin variants of each participants were determined using cellulose acetate electrophoresis according to previously published protocols [8] on the zeroth day of donor's blood collection. For each electrophoretic run, combination of hemolysate from a sickle cell trait (AS), Hb SC trait, and foetal blood (to provide Hb F) samples served as the control. As this assay is not genomic assay, the reporting of the results was based on only the bands detected [9].

#### 2.4.5. Osmotically Induced Red Cell Haemolysis Test

Osmotic-induced red cell haemolysis was determined on the donor's blood on the seventh, fourteenth, twenty-first, and thirty-fifth days of blood collection. In accordance with the previously published protocol [10], 50 *μ*L of participant's washed red blood cells was introduced to three different concentrations of buffered sodium chloride (0.85, 0.5, and 0.05%). After centrifugation at 1200 g for 5 minutes, the percent of osmotically induced haemolysis was assayed spectrophotometrically using UV Mini-1240 (Shimadzu Europa, China) at 540 nm. The osmotic-induced red cell lysis was calculated as follows: percent haemolysis = 100 [(*A*(0.5%) − *A*(0.85%))/(*A*(0.05%) − *A*(0.85%))], where *A* (0.5%), *A* (0.05%), and *A* (0.85%) are the absorbances at NaCl concentrations 0.5%, 0.05%, and 0.85%, respectively.

#### 2.4.6. Percentage Foetal Haemoglobin (%Hb F)

In accordance with previously described protocols [11], %Hb F concentration was estimated for each sample on the zeroth day of blood collection. Samples were centrifuged at 2000g for 10 minutes, and packed RBCs were washed three times with isotonic saline to remove plasma before the hemolysate was prepared. The absorbances were read using UV Mini-1240 (Shimadzu Europa, China) at 540 nm wavelength. The proportion of Hb F was calculated from the absorbance ratio between Hb F and total Hb after correcting for the dilution factor as follows: Hb F (%) = [(100*∗*absorbance of test)/(absorbance of ref*∗*20)].

#### 2.4.7. Free Haemoglobin (Hb) Estimation

Free Hb was estimated on the seventh, fourteenth, twenty-first, and thirty-fifth days of donor's blood collection. Plasma haemoglobin was estimated using the Mindray 3500 automated haematology analyser (Mindray, China). Percentage spontaneous haemolysis was calculated using the formula used by Sawant [12]: (% haemolysis =  (100 − HCT)*∗* plasma haemoglobin(*g*/*dl*)/Total Hb (*g*/*dl*)), where Hct is the haematocrit of whole blood. The acceptable limit of % free haemoglobin was set at 0.8% in accordance with the previous recommendation [13].

#### 2.4.8. Plasma Potassium Ion (K+) Estimation

Plasma potassium ion for each sample was estimated using the Convergys ISE autoelectrolyte analyser (Convergent technologies, Germany) on the zeroth, seventh, fourteenth, twenty-first, and thirty-fifth days of donor's blood collection in accordance with the manufacturer's protocol. The potassium ion levels were recorded in mEq/L.

#### 2.4.9. Full Blood Count (FBC)

Full blood count for each sample was estimated using the Mindray 3500 automated haematology analyser (Mindray, China). The RBC count, red cell indices, haemoglobin (Hb) concentration, platelet count, and WBC count and WBC absolute differential counts were recorded. The FBC was recorded on days 0 (baseline), 7, 14, 21, and 35 of storage.

### 2.5. Data Analyses

Data collected were entered into Microsoft Office Excel 2013 and analysed using GraphPad Prism version 8.0.2 for Windows (GraphPad Co., USA). The data were explored for normality using the D'Agostino test. As the data were not normally distributed, significant differences between repeated measurements (days 0, 7, 14, 21, and 35) were undertaken using Friedman's repeated measure test; post-hoc analyses were undertaken using Dunn's test correction. All three sample comparisons for non-repeated measurements were undertaken using the Kruskal–Wallis test. Moreover, all two sample comparisons were undertaken using the Mann–Whitney test. Statistical significance was set as *p* < 0.05 under the two-tailed assumption.

## 3. Results

The demographic and clinical data of the blood donors are recorded in Table 1. Majority (87.4%) of the blood donors were teenagers. Overwhelmingly, the blood donors were predominantly males (95.1% vs 4.9% females). Also, blood group O was the most represented blood group. Furthermore, whereas 26.2% had inherited haemoglobin variants, 24.3% had qualitative G6PD defect. Additionally, majority of the blood donors had elevated foetal haemoglobin levels (%Hb F > 2.5).

The data were explored for how inherited haemoglobin variants impacted changes in plasma K+, free plasma haemoglobin, and osmotically-induced red cell fragility during storage (Table 2). Plasma K+ levels significantly increased over the 35 days storage (compared with baseline) in all participants irrespective of inherited haemoglobin variants (*p* < 0.0001 in each case; by means of Friedman's repeated measure test). However, when the daily plasma K+ levels were compared across the inherited haemoglobin variants, Hb AC had comparatively non-statistically significant higher median plasma K+ levels (*p* > 0.05; by means of the Kruskal–Wallis test). Additionally, irrespective of the haemoglobin type, osmotically induced haemolysis significantly decreased during the 35 days of storage. However, when the weekly estimates of osmotic-induced haemolysis were compared across the inherited haemoglobin variants, units from Hb AC donors had significantly lower median osmotic-induced haemolysis compared with Hb A or Hb AS (*p* < 0.0001; by means of Kruskal-Wallis test). Furthermore, the median of the spontaneously released haemoglobin increased with storage irrespective of the inherited haemoglobin type (*p* < 0.05 in each case by means of Friedman repeated measure test). Supplementary figure S1 (D, E, and F) each gives a visual plot of the changes with respect to haemoglobin type.

The impact of blood donors' qualitative G6PD status on whole blood storability was explored (Table 3). The median plasma K+ levels of donor blood increased during the 35 days' storage irrespective of G6PD status (*p* < 0.0001 in each case by means of Friedman's repeated measure test). However, when the daily median plasma K+ levels were compared across the G6PD status, G6PD normal individuals had comparatively non-statistically significant higher median plasma K+ levels. Also, the osmotic-induced haemolysis of donor blood units reduced during storage irrespective of the G6PD status (*p* < 0.0001 in each case). However, when the weekly median plasma K+ levels were compared across the G6PD status, G6PD normal individuals had comparatively higher median plasma K+ levels that reached statistical significance on day 35 (*p* = 0.043; by means of the Mann–Whitney test). Furthermore, the % free haemoglobin significantly increased during storage irrespective of qualitative G6PD status, with a trend towards nonstatistically significant increased levels in G6PD deficient units. Supplementary figure S1 (A, B, and C) each gives a visual plot of the changes with respect to G6PD status of donated blood unit.

When the blood donor units were stratified per the participant age group (Table 4), there were significantly increased median plasma K+ levels (*p* < 0.0001) and free haemoglobin levels (*p*=0.0079) during the 35 days storage irrespective of the age group; osmotic-induced red cell lysis however decreased during storage irrespective of the age group. When the estimates of storage lesions were compared across the age group per the respective weekly measurements, donor units from participants ≥20 years generally had higher estimates; however, only free haemoglobin levels significantly differed on day 21 (*p*=0.0078 by means of the Mann–Whitney test).

The data were also interrogated per the blood group of the donor unit (Table 5). Generally, the same trend of changes in plasma K+, osmotic fragility, and free haemoglobin was recorded per each blood group. Even though blood group B donor units had comparatively higher daily median estimates of plasma K+, osmotic fragility, and free haemoglobin, none of these reached statistical significance. When the data were stratified based on %Hb F levels, there were comparable levels of plasma K+, free haemoglobin, and osmotic-induced haemolysis between donor units with elevated and normal %Hb F levels (supplementary data S1)

The data were further explored for percentage median changes in the plasma K+ per donor characteristics (Table 6). Donor units that had defective G6PD status had comparatively lower median %changes in plasma K+ levels during the 35 days' storage. Also, in relation to haemoglobin type of donor units, participants with Hb AC had comparatively higher median %change in plasma K+ levels during storage, except on day 35 where Hb AS donor units had the highest median % change in plasma K+ levels. In relation to the age group, donor units from participants ≥20 years had comparatively higher % median change in plasma K+ levels. Furthermore, in relation to the ABO blood group, donor units with blood group B had comparatively higher % median change in plasma K+ levels.

## 4. Discussion

It is generally recognized that blood units collected from different donors but banked in the same anticoagulant and stored for the same number of days may show differing levels of storage lesion [14,15]. This is indicative that inherent donor characteristics influence the degree of blood donor storage lesion. In this cohort study, we sought to explore the potential impact of inherited G6PD status, haemoglobin variants, blood group, and donor age on some parameters of storage lesion of whole blood units banked at 4°C in CPDA-1 for 35 days. Our study found that, whereas osmotic-induced red cell lysis was significantly lower in haemoglobin AC donor units (compared to haemoglobin A, and AS units) at all time points, it was significantly higher on day 35 in individuals with G6PD defect (when compared with G6PD normal donor units). Additionally, during the 35 days' storage, the percentage change in median plasma potassium levels was comparatively higher in G6PD normal, haemoglobin AC, donors aged ≥20 years, and blood group B donor units.

Our previous work showed that 19.5% of blood donors in the Brong-Ahafo region, Ghana, carried the sickle cell trait. However, we did not quantify the impact of this high incidence of structural haemoglobin variants on storability of whole blood. Herein, we show that donor units from individuals with Hb AC showed significantly lower osmotic-induced haemolysis compared with Hb AS or Hb A donor units. Additionally, Hb AC donor units demonstrated higher plasma K+ levels and higher percentage change in K+ levels at each point of measurement (day 7, 14, 21, or 35) when compared with Hb A or AS donor units. It should be noted that at 35 days in storage, only the Hb AS donor units had percentage free haemoglobin below the acceptable 0.8% threshold [13] compared with Hb A or AC units; each of these units had % free haemoglobin above the acceptable limits. This level of plasma K+ and % free haemoglobin in Hb AC units are indicative of poor storability of such units. Previously, Ould Amar et al. also reported comparatively higher plasma K+ levels as well as significantly reduced osmotic fragility of Hb AC donor units stored in SAG-mannitol for 42 days [16]. Although that study used packed red cells and SAG-mannitol as the anticoagulant compared with whole blood units and CPDA-1 anticoagulant in the present study, the consistency in the results are indicative that these differences in storability of donor units with different haemoglobin variants are not artefact. There is however paucity of data regarding the impact of haemoglobin AC on storability of red blood cells. Considering that 9.7% of the blood donors in the studied area inherited the Hb AC trait, more studies are required to properly characterize these donor units. With regards Hb AS, other previous studies have also reported reduced elastic properties [17] and poor efficiency of leuko-filtration [18] of Hb AS donor units when compared with Hb A donor units. Taken together, the key question that remains to be critically delineated is the clinical implications of transfusing Hb AC or Hb AS donor blood units. For example, will the significantly reduced osmotic-induced haemolysis of Hb AC units translate into ability of such units to withstand post-transfusion milieu better than other donor units with different haemoglobin types? These will require well-controlled in situ clinical trials that take into consideration donor-to-donor variables vis-a-vis recipients' pathology, and subsequent follow up on recipients of these donor units using high-throughput techniques to comprehensively elucidate posttransfusion outcomes since in vitro variability in storage lesions recorded in donor units with inherited haemoglobin variants may not necessarily translate into inferior transfusion outcomes.

Our previous study estimated the qualitative G6PD defect to be 19.5% among blood donors in the Brong-Ahafo region of Ghana [4]. Taken together with the fact that the World Health Organization estimates 15–26% prevalence of G6PD deficiency in Ghana, a substantial proportion of donor unit carries this red cell enzymopathy. Previously, it was shown that G6PD deficient (Class II Mediterranean variant) donor units showed a trend of lower spontaneously free haemoglobin as well as significantly higher plasma potassium over 42 days of storage [19]. These findings are not consistent with the findings presented herein since we report comparable plasma K+ levels between G6PD deficient and G6PD normal units over 35 days. Again, whereas we found a trend of higher median free haemoglobin in G6PD-deficient units throughout storage, Tzounakas et al. reported lower free haemoglobin during storage. In reconciling these disparate findings, it is important to note that, whereas G6PD status was determined qualitatively in the present study, genotyping was used in Tzounakas' work. Therefore, the donor units classified as G6PD-deficient in the present study may presumably be class IIIA-variant (known to be associated with 10–60 enzyme activity compared to <10% activity in G6PD II variant) which is prevalent in people of African descent, and these differences in G6PD genotype might have introduced variability in the results. This hypothesis is corroborated by a previous study that found no significant differences in supernatant haemoglobin, plasma K+, and blood pH between G6PD deficient and G6PD normal donors of African descent [20]. Taken together with the fact that blood donation rate in most sub-Saharan African countries falls short of basic requirements and that about one-in-twenty donors are G6PD deficient, donor units will inadvertently contain G6PD-deficient units. Nevertheless, in view of the demonstrable reduced posttransfusion efficacy of G6PD deficient units [21,22], there needs be incorporation of G6PD screening into the predonation screening algorithm in areas where G6PD is prevalent, and subsequent labelling of such donor units to ensure that susceptible recipients are protected in accordance with WHO recommendations [23].

Furthermore, in accordance with the previous studies [24,25], this study found that younger age of donor was associated with better storability of whole blood units as determined by in-bag haemolysis and plasma K+ levels. Additionally, we report herein that storage lesions as measured by plasma K+ levels and free haemoglobin were comparably higher in blood group B donor units compared with other blood units. Although the observed differences did not reach statistical significance, we propose that any future prospective studies seeking to understand storability of donor blood units should take the blood group into consideration so as to account for any possible confounding effects from that angle.

Limitations of the present study include our inability to estimate quantitatively the G6PD status and/or G6PD genotype of the blood donor units. The estimation of enzymatic activity of the G6PD enzyme of the donor red blood cells would have provided a means of stratification based on enzyme activity to provide a more in-depth analysis. Also, although subclinical malaria parasitaemia may be present in some of the donors, we did not estimate malaria parasitaemia in donated blood units. It is plausible that malaria parasitaemia could have also impacted the storage lesions. Moreover, the inheritance of the thalassaemia trait has been demonstrated to affect K+ homeostasis [26] and osmotic fragility [27]. The thalassaemia trait is among the haemoglobinopathies that have been shown to have coevolved with *Plasmodium falciparum* infection and will likely be prevalent in the studied population. However, this study did not evaluate for the inheritance of thalassaemia in the blood donor units and could therefore not account for any such potential confounding effects. In spite of these limitations, we believe our present study highlightsthat inherited red cell enzymopathy, haemoglobin variant, and blood group or age blooddonor are important considerations when accounting for donor-to-donor variabilityin storability of whole blood. 

## Figures and Tables

**Table 1:** Demographic and clinical variables of blood donors.

Variable	Frequency	%
*Age (years)*		
16–19	90	87.4
20–29	11	10.7
>29	2	1.9

*Sex*		
Male	98	95.1
Female	5	4.9

*Blood group*		
A	20	19.4
B	24	23.3
AB	1	0.9
O	58	56.3

*Haemoglobin type*		
A	76	73.8
AC	10	9.7
AS	16	15.5
SC	1	1.0

*Qualitative G6PD status*		
Normal	78	75.7
Full defect	24	23.3
Partial defect	1	1.0

*%Hb F*		
<2.5	24	23.3
≥2.5	79	76.7

Hb F: foetal haemoglobin; G6PD: glucose 6 phosphate dehydrogenase enzyme.

**Table 2:** Stratification based on inherited haemoglobin variants of donor blood unit.

Variable	Days					*p* value
	Day 0	Day 7	Day 14	Day 21	Day 35	
*Plasma K+ levels*						
Hb A	4.040	10.040	14.680^†^	17.620^†^	23.760^†^	<0.0001
Hb AC	4.140	10.480	15.330^†^	17.970^†^	25.440^†^	<0.0001
Hb AS	4.040	9.840	14.200^†^	16.750^†^	23.870^†^	<0.0001
*p* value	ns	ns	ns	ns	ns	

*Osmotic-induced lysis*						
Hb A	ND	82.980	55.690^†^	40.550^†^	53.120^†^	<0.0001
Hb AC	ND	26.820	12.210^†^	11.110^†^	16.870^†^	0.0083
Hb AS	ND	73.720	40.120^†^	31.430^†^	45.970^†^	<0.0001
*p* value		**<0.0001**	**<0.0001**	**<0.0001**	**<0.0001**	

*% free haemoglobin*						
Hb A	ND	0.000	0.000	0.609^†^	0.910^†^	<0.0001
Hb AC	ND	0.000	0.281	0.323^†^	0.914^†^	0.0015
Hb AS	ND	0.000	0.197	0.275	0.762^†^	0.018
*p* value		ns	ns	ns	ns	

ND means not determined; ns means not significant; ^†^median value significantly differed from day 0 (K+ levels), or day 7 (for osmotic fragility or % free haemoglobin); ns means not statistically significantly different; statistical significance was estimated per haemoglobin variant in relation to days of storage by means of Friedman's repeated measure test with Dunn's multiple correction; across the three haemoglobin variants, statistical differences within each day were calculated using the Kruskal–Wallis test.

**Table 3:** Stratification based on the qualitative G6PD status.

Variable	Days					*p* value
	Day 0	Day 7	Day 14	Day 21	Day 35	
*Plasma K+ levels*						
G6PDd	4.050	9.720	13.750^†^	16.710^†^	23.820^†^	<0.0001
G6PD normal	4.040	10.200^†^	14.940^†^	17.650^†^	23.830^†^	<0.0001
*p* value	ns	ns	ns	ns	ns	

*Osmotic-induced lysis*						
G6PDd	ND	74.940	37.060^†^	27.640^†^	42.340^†^	<0.0001
G6PD normal	ND	81.940	54.930^†^	40.400^†^	50.880^†^	<0.0001
*p* value		ns	ns	ns	**0.0438**	

*% free haemoglobin*						
G6PDd	ND	0.000	0.000	0.6440^†^	0.980^†^	<0.0001
G6PD normal	ND	0.000	0.000	0.590^†^	0.880^†^	<0.0001
*p* value		ns	ns	ns	ns	

ND means not determined; ns means not significant; ^†^median value significantly differed from day 0 (K+ levels), or day 7 (for osmotic fragility or % free haemoglobin); ns means not statistically significantly different; statistical significance was estimated per each G6PD status in relation to days of storage by means of Friedman's repeated measure test with Dunn's multiple correction; across the two G6PD stratification, statistical differences within each day were calculated using the Mann–Whitney test.

**Table 4:** Stratification based on the age group.

Variable	Days					*p* value
	Day 0	Day 7	Day 14	Day 21	Day 35	
*Plasma K+ levels*						
16–19 yrs	4.035	9.995^†^	14.680^†^	17.410^†^	23.55^†^	**<0.0001**
≥20 yrs	4.180	10.890	15.250^†^	20.130^†^	25.780^†^	**<0.0001**
*p* value	ns	ns	ns	ns	ns	

*Osmotic-induced lysis*						
16–19 yrs	ND	79.570	50.500^†^	38.060^†^	48.550^†^	**<0.0001**
≥20 yrs	ND	89.780	55.810^†^	38.440^†^	56.940^†^	**0.0079**
*p* value		ns	ns	ns	ns	

*Free haemoglobin*						
16–19 yrs	ND	0.000	0.000	0.5800^†^	0.900^†^	**<0.0001**
≥20 yrs	ND	0.000	0.5590	0.7190^†^	0.811^†^	**0.0002**
		ns	ns	**0.0078**	ns	

ND means not determined; ns means not significant; ^†^median value significantly differed from day 0 (K+ levels), or day 7 (for osmotic fragility or % free haemoglobin); ns means not statistically significantly different; statistical significance was estimated per each age group in relation to days of storage by means of Friedman's repeated measure test with Dunn's multiple correction; across the two age groups, statistical differences within each day was calculated using the Mann–Whitney test.

**Table 5:** Stratification of data per blood group.

Variable	Day					*p* value
	Day 0	Day 7	Day 14	Day 21	Day 35	
*Plasma K+ levels*						
Group A	3.990	10.020	14.790^†^	16.490^†^	22.840^†^	<0.0001
Group B	4.035	10.180	15.240^†^	18.250^†^	24.920^†^	<0.0001
Group O	4.070	10.020^†^	14.080^†^	17.460^†^	23.930^†^	<0.0001
*p* value	ns	ns	ns	ns	ns	

*Osmotic-induced lysis*						
Group A	ND	78.840	50.480^†^	36.700^†^	49.660^†^	0.0003
Group B	ND	80.860	45.400^†^	39.740^†^	51.930^†^	<0.0001
Group O	ND	79.360	54.480^†^	37.780^†^	47.750^†^	<0.0001
*p* value		ns	ns	ns	ns	

*Free haemoglobin*						
Group A	ND	0.000	0.000	0.316	0.851^†^	<0.0001
Group B	ND	0.000	0.212	0.609^†^	0.912^†^	<0.0001
Group O	ND	0.000	0.000	0.614^†^	0.891^†^	<0.0001
*p* value		ns	ns	ns	ns	

ND means not determined; ns means not significant; ^†^median value significantly differed from day 0 (K+ levels), or day 7 (for osmotic fragility or % free haemoglobin); ns means not statistically significantly different; statistical significance was estimated per each blood group in relation to days in storage by means of Friedman's repeated measure test with Dunn's multiple correction; across the two age groups, statistical differences within each day were calculated using the Kruskal–Wallis test.

**Table 6:** Median changes in plasma K+ levels in donor units during 35 days' storage.

Variables	ΔK+ (%ΔK+) plasma levels			
	Day 7	Day 14	Day 21	Day 35
*G6PD status*				
Normal	6.165 (150.4)	10.99 (269.1)	13.730 (336.8)	19.800 (487.1)
Defective	5.640 (135.0)	9.690 (240.5)	12.810 (312.1)	19.91 (469.6)

*Haemoglobin type*				
Hb A	5.990 (149.8)	10.620 (259.5)	13.630 (336.8)	19.830 (480.3)
Hb AC	6.330 (157.7)	11.220 (267.8)	13.910 (350.4)	21.120 (489.5)
Hb AS	5.910 (150.8)	10.250 (251.8)	12.740 (318.9)	19.640 (491.0)

*Age group (years)*				
<20	5.975 (147.5)	10.750 (260.4)	13.430 (329.9)	19.430 (484.9)
≥20	6.700 (150.0)	11.060 (264.0)	15.250 (339.2)	21.800 (484.6)

*Blood group*				
A	6.040 (162.8)	10.790 (260.6)	12.400 (325.1)	19.070 (475.3)
B	6.290 (162.0)	11.170 (282.4)	14.160 (351.6)	21.300 (520.3)
O	5.875 (144.1)	10.190 (244.4)	13.320 (319.6)	19.890 (471.9)

Change in potassium was calculated as difference between the daily measurement and the baseline (day 0) K+ measurement.

## Data Availability

The dataset used during the current study is available from the corresponding author on reasonable request.

## Abbreviations

Hb AS: Sickle cell trait
G6PDd: Glucose 6-phosphate dehydrogenase deficiency
Hb AC: Haemoglobin C trait
CPDA-1: Citrate phosphate dextrose adenine 1.

## Supplementary Materials

Supplementary Materials Supplementary figure S1: line graph showing the changes in storage lesions with respect to days in storage. Supplementary file S2: stratification of donor units based on %Hb F levels.
