# Supplementary material for: Blood Donors' Age, Haemoglobin Type, G6PD Status, and Blood Group Impact Storability of CPDA-1 Banked Whole Blood: A Repeated-Measure Cohort Study in Cape Coast, Ghana
Source: Adv Hematol. 2020 May 30;2020:4959518. doi: 10.1155/2020/4959518 (PMC7277056; doi:10.1155/2020/4959518)
Supplement: Supplementary Materials — Supplementary figure S1: line graph showing the changes in storage lesions with respect to days in storage. Supplementary file S2: stratification of donor units based on %Hb F levels. [file 4959518.f1.pdf]

## Supplementary data

### Supplementary figure S1: Line graph showing the changes storage lesions with respect to days in storage

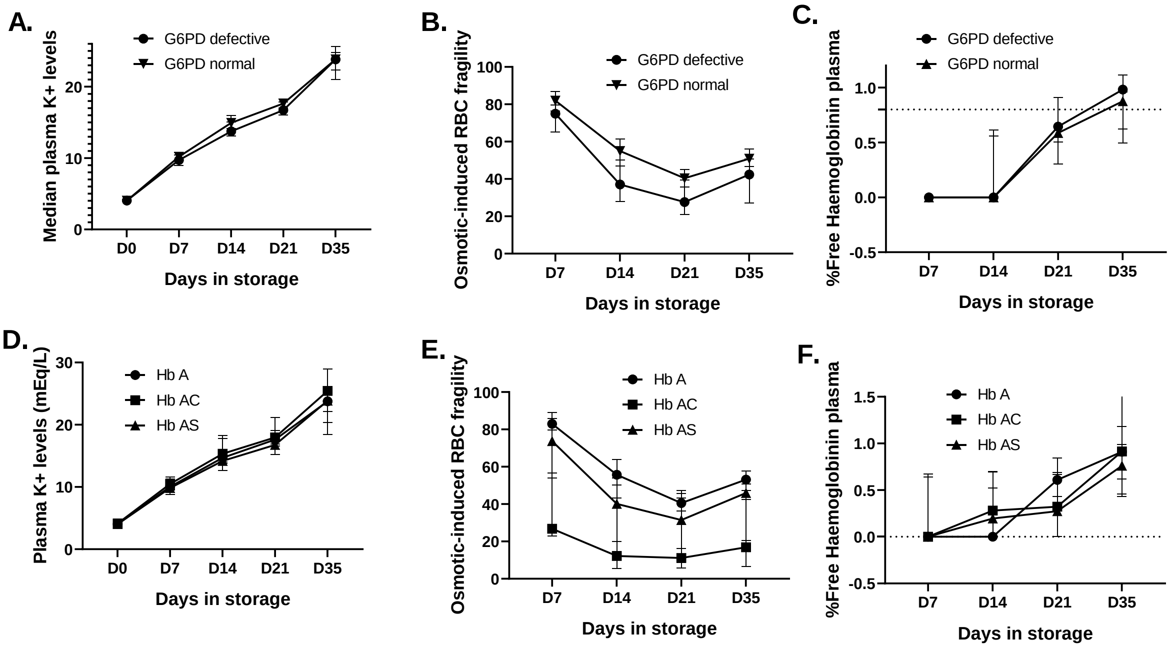


**Supplementary figure S1: Line graph showing the changes storage lesions with respect to days in storage.** Figures A, B, and C each depicts storage lesions quantified by changes in plasma potassium (A), osmotic-induced red cell fragility (B) and %free plasma haemoglobin (C) stratified per G6PD status of donated blood units. D, E and F each depicts storage lesions quantified by changes in plasma potassium (D), osmotic-induced red cell fragility (E) and %free plasma haemoglobin (F) stratified per inherited haemoglobin type of donated blood units. All figures are plotted using median with 95% confidence intervals.

### Supplementary file S2: Stratification of donor units based on %Hb F levels.

| **Variable** | | | **Day** | | | **P-value** |
|---|---|---|---|---|---|---|
| | Day 0 | Day 7 | Day 14 | Day 21 | Day 35 | |
| **Plasma K+ levels** | | | | | | |
| %Hb F<2.5 | 4.050 | 10.110 | 14.200† | 17.600† | 23.990† | <0.0001 |
| %Hb F≥2.5 | 4.030 | 10.020† | 14.740† | 17.430† | 23.690† | <0.0001 |
| p-value | ns | ns | ns | ns | ns | |
| **Osmotic-induced lysis** | | | | | | |
| %Hb F<2.5 | ND | 79.770 | 40.260† | 28.390† | 48.340† | <0.0001 |
| %Hb F≥2.5 | ND | 79.620 | 53.960† | 39.810† | 49.070† | <0.0001 |
| p-value | | ns | ns | ns | ns | |
| **Free haemoglobin** | | | | | | |

| | | | | | | |
|---|---|---|---|---|---|---|
| %Hb F<2.5 | ND | 0.000 | 0.000 | 0.3990 | 0.901† | <0.0001 |
| %Hb F≥2.5 | ND | 0.000 | 0.000 | 0.619† | 0.857† | <0.0001 |
| p-value | | ns | ns | ns | ns | |

Hb F = fetal haemoglobin; ND means not determined; ns means not significant; † indicates that median value significantly differed from day 0 (K+ levels), or day 7 (for osmotic fragility or %free haemoglobin); "ns" means not statistically significantly different; Statistical significance was estimated per each %Hb F category in relation to days of storage by means of Friedman's repeated measure test with Dunn's multiple correction; Across the two %Hb F stratification, statistical differences within each day was calculated using Mann-Whitney test.
